# COVID-19 Perceptions Among Communities Living on Ground Crossings of Somali Region of Ethiopia: Community Cross-Sectional Survey Study

**DOI:** 10.2196/66751

**Published:** 2025-04-24

**Authors:** Alinoor Mohamed Farah, Abdifatah Abdulahi, Abdulahi Hussein, Ahmed Abdikadir Hussein, Abdi Osman, Mohamed Mohamud, Hasan Mowlid, Girum Hailu, Fathia Alwan, Ermiyas Abebe Bizuneh, Ahmed Mohammed Ibrahim, Elyas Abdulahi

**Affiliations:** 1 Faculty of Public Health, Institute of Health, Jigjiga University, Kebele 16, Jigjiga, Ethiopia, 251 911053913; 2 Faculty of Medical Sciences, Institute of Health, Jigjiga University, Jigjiga, Ethiopia; 3 Health and Development Division, Intergovernmental Authority on Development, Addis Ababa, Ethiopia; 4 Research and Community Service Directorate, Jigjiga University, Jigjiga, Ethiopia

**Keywords:** COVID-19, perceptions, border-crossings, SARS-CoV-2, coronavirus, respiratory, infectious, pandemic, Somali, Ethiopia, Africa, community-based, World Health Organization, WHO, cross-sectional study, multistage sampling technique, transmission, cross-border, community engagement

## Abstract

**Background:**

The COVID-19 pandemic has profoundly affected the movement of people across borders in Eastern and Southern Africa. The implementation of border closures and restrictive measures has disrupted the region’s economic and social dynamics. In areas where national authorities lack full control over official and unofficial land crossings, enforcing public health protocols to mitigate health risks may prove challenging.

**Objective:**

This study aimed to assess perceived factors that influence the spread and control of COVID-19 among Somali communities living on and near ground crossings in Tog Wajaale, Somali region, Ethiopia.

**Methods:**

A community-based cross-sectional study was conducted using a multistage sampling technique. Beliefs and perceptions of the virus’s spread and control were partially adapted from the World Health Organization (WHO) resources, exploring four main perception themes: (1) perceived facilitators for the spread of the virus, (2) perceived inhibitors, (3) risk labeling, and (4) sociodemographic variables. A sample size of 634 was determined using the single proportion formula. Standardized mean scores (0‐100) and SDs categorized perception themes, with mean differences by sociodemographic variables analyzed using ANOVA and *t* tests. Statistical significance was established with a 95% CI and a *P* value below .05. The data were analyzed using STATA version 14.1.

**Results:**

Factors influencing COVID-19 spread and control include behavioral nonadherence and enabling environments. A total of 81.9% (439/536) did not comply with social distancing, and 92.2% (493/536) faced constraints preventing them from staying home and enabling environments. Misconceptions were prevalent, including beliefs about hot weather (358/536, 66.8%), traditional medicine (36/536, 6.7%), and religiosity (425/536, 79.3%). False assurances also contributed, such as feeling safe due to geographic distance from hot spots (76/536, 14.2%) and perceiving the virus as low-risk or exaggerated (162/536, 30.2%). Only 25.2% (135/536) followed standard precautions and 29.9% (160/536) were vaccinated. Employment, region, income, sex, education, and information sources significantly influenced behavioral nonadherence, myth prevalence, and false assurances.

**Conclusions:**

The findings highlight the need for substantial risk communication and community engagement. Only 46.6% (250/536) of individuals adhered to precautionary measures, there was a high perception of nonadherence, and essential COVID-19 resources were lacking. Additionally, numerous misconceptions and false reassurances were noted. Understanding cross-border community behavior is crucial for developing effective, contextually appropriate strategies to mitigate COVID-19 risk in these regions.

## Introduction

Cross-border movement has been identified as an important factor in COVID-19 transmission in Eastern and Southern Africa, and most countries in the region have restricted entry to reduce virus importation. However, the region’s economy and social fabric are dependent on the cross-border movement of goods and people, and it was anticipated that border closures would have the same, if not greater, impact than COVID-19 [1,2]. As a result, countries have relaxed COVID-19 containment measures and lifted border closures, increasing the risk of the virus spreading internationally.

Under normal circumstances, a diverse range of people crosses national borders in the Eastern and Southern Africa region to participate in vital activities. Truck and boda-boda (motorcycle taxi) drivers, small- and large-scale traders, tourists, migrant workers, fisherfolk and pastoralists, refugees, returnees, border community members, and people traveling to seek health care and other social services are among those affected. These people interact with the communities they pass through on their way to and from work. Border officials, security officers, and support staff interact with travelers daily, increasing the risk of community spread [2-6].

Since the International Health Regulations 2005 came into effect in 2007, there has been growing recognition that, unlike airports and ports, ground crossings frequently represent informal passages between 2 countries with no physical structure, barriers, or borders. Furthermore, ground crossing is important for the international spread of diseases. Travelers and those who live and work on or near borders are particularly vulnerable to this threat [7].

Communities that live on and near ground crossings vary in size and density. Cross-border movement is a daily necessity for many people living in these communities for work, trade, family visits, schooling, health care services, religious activities, entertainment, and other reasons. However, health measures to control public health risks may be difficult to implement in places where national authorities are unable to fully monitor formal and informal ground crossings [7].

Given that borders have already been reopened, it is critical to understand cross-border transmission dynamics, movement patterns, and the behavior of cross-border actors to develop effective, contextually appropriate risk communication and community engagement (RCCE) strategies to mitigate COVID-19 risk. Thus, this study aimed to assess the perceived factors that influence the spread and control of COVID-19 among Somali communities living on and near ground crossings.

## Methods

### Study Design, Setting, and Period

A community-based cross-sectional study was conducted from December 25 to 31, 2022, in Tog Wajaale, Somali region, Ethiopia. Tog Wajaale, also known as Wajaale, is a city in the Somali region near the border with Somaliland. Tog Wajaale is the main border crossing for goods entering and leaving Somaliland, primarily from Berbera, the country’s main port. Tog Wajaale is located on the border between Ethiopia and Somaliland. It is 70 km east of Jigjiga, the capital of the Somali region.

### Populations

All adult populations older than 18 years were included in the source population. The study participants were all adult populations older than 18 years who had lived in the Tog Wajaale town for at least 6 months.

### Eligibility Criteria

The study included individuals aged 18 years and older who had resided in the study area for a minimum of 6 months. Those who were experiencing mental illness or severe health issues during the study period were excluded from participation.

### Sample Size Determination

The sample size was obtained using the single proportions formula with the specified parameters of 50% (*P*=.50, as there is no comparable study in Ethiopia), 95% CI (*Z*_1_ – *α*/2) 1.96%, and 5% margin of error (*d*). Adding a 10% nonresponse rate and a design effect of 1.5 resulted in a required sample size of 634.

### Sampling Technique

A multistage sampling technique is used to select participants for the study. In the first stage, 1 city council in the region, based on the border with the neighboring country and demographic diversity, is selected. In the second stage, 5 kebeles (subdistricts) are randomly selected, and the total sample size is allocated by probability proportional to size (PPS) to each kebele based on the number of registered households. In the third stage, using the list of households as the sampling frame, a systematic random sampling technique is used to select the study participants from the kebeles.

### Data Collection and Quality Control

Data were collected using a pretested structured questionnaire. Ten BSc nurses fluent in the local language collected data through face-to-face interviews at the household level. Beliefs and perceptions of the virus’s spread and control were partially adapted from the World Health Organization (WHO) resources. Specifically, some of the questions were adapted from the COVID-19 rapid quantitative assessment tool [8,9]. Three main perception themes were explored: perceived facilitators for the spread of the virus (6 items), perceived inhibitors (7 items), and risk labeling (8 items), as well as sociodemographic variables, including access to communication resources. Prior to participation, the household head or any individual over 18 years of age was fully informed of the study details and asked for consent. Questionnaires were reviewed daily for completeness, and respondents were contacted to see if any information was missing. The investigators regularly supervised the data collection process. To ensure data quality, ongoing supervision and follow-ups were conducted throughout the collection period.

### Data Analysis

KoBoToolbox, an open-source data collection platform, was used to collect data. KoBo-collected responses were initially encoded in a Microsoft Excel database and then exported to STATA version 14.1 for analysis. The frequency tables were used to present the respondents’ background variables as well as specific belief items. Standardized mean scores (0‐100) and SDs were used to describe the lists of factor categories based on the perception themes. To compare mean differences by sociodemographic variables, 1-way ANOVA and *t* tests were used. A factor loading score of 0.4 was used as a cutoff value to retain items in each category [10]. Exploratory factor analysis (EFA) categorized perceptions into 4 themes: facilitators of virus transmission (6 factors), inhibitors (7 factors), risk identification (8 factors), and sociodemographic factors, including communication resource access. The sample’s adequacy for EFA was confirmed by the Kaiser-Mayer-Olkin measure (KMO>50%). Statistical significance was determined using a 95% CI and a *P* value below .05.

### Operation Definition and Variables

The *perceived factors* influencing the spread and control of COVID-19 include exacerbating factors, inhibiting factors, and risk labeling.

#### Perceived COVID-19 Exacerbating Factors

##### Behavioral Nonadherence

Noncompliance with public health measures designed to control the spread of COVID-19 encompasses various behaviors exhibited by individuals or groups. These actions diverge from the recommended guidelines and may include disregarding social distancing protocols, neglecting to use face masks, refusing COVID-19 vaccination, or displaying discriminatory attitudes toward others in relation to the pandemic.

##### Lack of Enabling Environment

The absence of essential conditions that support the implementation of public health measures to prevent COVID-19 transmission. For example, economic and social constraints that force people to leave their homes for work or social issues; overcrowded living and working conditions that may not allow physical distancing; lack of access to personal protective equipment such as face masks; and insufficient availability of clean water, soap, hand rub alcohol, or sanitizers.

### Perceived COVID-19 Inhibiting Factors

#### False Assurance

Misconceptions or preconceptions that diminish the perceived threat of COVID-19 frequently result in a relaxed compliance with preventive protocols. Examples include the belief that one’s location is far removed from epidemic centers or the underestimation of the seriousness of the illness.

#### Myths

Misinformed or culturally rooted beliefs that influence public perception of COVID-19 and its risks often undermine scientific evidence. Examples include beliefs that hot weather kills the virus, reliance on traditional medicine as a cure, or attributing protection to religious practices alone.

#### Standard Precautions

The actions taken by individuals to minimize the risk of COVID-19 transmission were consistent with the recommended public health guidelines. These include wearing masks, maintaining hand hygiene, avoiding crowded areas, and vaccinating against COVID-19.

### Risk Labeling

Risk labeling involves perceiving a specific sect of the community to be at a high risk of contracting COVID-19, especially older adults and people with underlying medical conditions.

### Ethical Considerations

This study was conducted in accordance with the guidelines of the Declaration of Helsinki, and all procedures involving research study participants were approved by the Jigjiga University institutional review board (RERC/052/2022). Written informed consent was obtained from all the participants. Participants were not compensated for participating in the study. The study participants’ data are confidential and protected.

## Results

### Sociodemographic Characteristics of Participants

The survey questionnaire was completed by 536 participants, resulting in a response rate of 84.5% (536/634). Table 1 summarizes the demographic information of the survey respondents. The majority of respondents (439/536, 81.9%) were younger than 40 years and male (313/536, 58.4%). Most of the respondents (153/536, 28.5%) had a high school education, followed by those who could read and write but with no formal education (114/536, 21.3%). The majority of participants in the study were married (335/536, 62.5%), ran their own businesses (261/536, 48.7%), were Muslim (501/536, 93.5%), and were of Somali origin (423/536, 78.9%). Furthermore, the majority of participants (287/536, 53.5%) used cellular data as their primary internet source, had an income greater than 5000 ETB (Ethiopian Birr; US $93) (324/536, 60.4%), and their primary source of information about COVID-19 was television (230/536, 42.9%).

**Table 1.** Sociodemographic characteristics of the respondents (N=536).

Characteristics		Values, n (%)
**Age (years)**		
	<40	439 (81.9)
	≥40	97 (18.1)
**Sex**		
	Male	313 (58.4)
	Female	223 (41.6)
**Education**		
	Cannot read and write	10 (1.9)
	Read and write	114 (21.3)
	Primary (grade 1‐8)	78 (14.6)
	High school (grade 9‐12)	153 (28.5)
	Diploma or level I-IV	104 (19.4)
	First degree (BSc or BA) and above	77 (14.4)
**Marital status**		
	Single (unmarried)	174 (32.5)
	Married	335 (62.5)
	Divorced	13 (2.4)
	Widowed	4 (0.7)
	Engaged (in relationship)	10 (1.9)
**Employment**		
	Private or business	261 (48.7)
	Daily laborer	75 (14)
	Government employee	80 (14.9)
	Student	34 (6.3)
	No job	86 (16)
**Religion**		
	Muslim	501 (93.5)
	Orthodox	27 (5)
	Protestant	8 (1.5)
**Place of origin**		
	Somali region	423 (78.9)
	Other regions	113 (21.1)
**Internet connection mostly used to get information about COVID-19**		
	Cellular data (telle)	287 (53.5)
	Wireless Wi-Fi	46 (8.6)
	Do not use internet	203 (37.9)
**Monthly income**		
	<5000 ETB^a^ (US $93)	212 (39.6)
	≥5000 ETB (US $93)	324 (60.4)
**Main source of information about COVID-19**		
	Television	230 (42.9)
	Radio	16 (3)
	Friends and neighbors	58 (10.8)
	Social media (Facebook, Twitter, YouTube, etc)	196 (36.6)
	Health workers	36 (6.7)
**Ever used the call center for information about COVID-19**		
	Yes	22 (4.1)
	No	514 (95.9)

a ETB: Ethiopian Birr.

### Perceived COVID-19 Exacerbating and Inhibiting Factors

Table 2 shows the prevalence of specific factors in the exacerbating and inhibiting categories. The prevalence of specific factors associated with behavioral nonadherence ranged from 81.9% (439/536) to 32.5% (174/536). The fact that people are still not adhering to physical or social distance accounted for a greater proportion of nonadherence. The magnitude of the absence of enablers that would support behavioral adherence ranged from 92.2% (494/536) to 5.4% (29/536). The fact that people could not stay home for economic and social reasons and living or working in overcrowded conditions contributed significantly to the lack of enablers. Environmental factors were perceived as more deterrent than behavioral nonadherence, indicating a strong tendency for externalizing factors that could exacerbate the spread of COVID-19 in the community.

**Table 2.** Perceived COVID-19 exacerbating and inhibiting factors (N=536).

Perceived factors			Descriptive statistics, n (%)
**Perceived COVID-19 exacerbating factors**			
	**Behavioral nonadherence**		
		People fear stigma and bias related to COVID-19	205 (38.3)
		People still do not respect social distance, ie, use crowded transportation means, still hug and shake each other’s hands while greeting	439 (81.9)
		People refused to take COVID-19 vaccine	174 (32.5)
	**Lack of enabling environment**		
		People do not stay at home for economic and social reasons	494 (92.2)
		People still live and work in a very crowded condition	403 (75.2)
		People do not have access to face masks, water, and sanitizers	29 (5.4)
**Perceived COVID-19 inhibiting factors**			
	**Myths**		
		The weather we live-in is too hot for coronavirus to survive	358 (66.8)
		We believe we have traditional medicine against COVID-19	36 (6.7)
		We are religious enough to control COVID-19	425 (79.3)
	**False assurances**		
		We live far away from COVID-19’s hot spot areas	76 (14.2)
		COVID-19 is not risky or exaggerated	162 (30.2)
	**Engaged in precaution**		
		Engaged in standard precaution measures of COVID-19	135 (25.2)
		Vaccinated against COVID-19	160 (29.9)

The most common myths were perceived as protectiveness from hot weather and religiosity, accounting for 66.8% (358/536) and 79.3% (425/536), respectively. Beliefs that the specific localities where respondents are currently living are far away from coronavirus hot spot areas and that COVID-19 is not dangerous or exaggerated contributed to 14.2% (76/536) and 30.2% (162/536) of the false assurances, respectively. On the other hand, the prevalence of people believing that the spread of COVID-19 would be controlled because of their active participation in standard precautionary measures and the COVID-19 vaccine were 25.2% (135/536) and 29.9% (160/536), respectively.

### Perceived Categories of COVID-19 Exacerbating and Inhibiting Factors

Table 3 presents the categories and lists of perceived COVID-19 exacerbations and inhibitors, along with their respective prevalence. EFA identified 2 major categories of perceived exacerbating and inhibiting COVID-19 factors in the target population. The first category of factors was classified as behavioral nonadherence, indicating noncompliance with recommended precautionary measures. Individuals engaging in handshaking, using crowded transportation, refusing COVID-19 vaccination, and exhibiting a factor loading score of 0.714 collectively contribute to behavioral nonadherence.

The lack of enabling environmental conditions, which are supposed to support the adaptation of precautionary measures, was the second category of exacerbating facilitating factors. With factor loading scores of 0.71, the lack of enablers was made up of economic reasons that challenged the stay-at-home principle, overcrowded living or working conditions, and a lack of personal protective equipment such as face masks and sanitizers. Behavioral nonadherence and lack of enabler factors explained 28.4% (152/536) of the variance in perceived virus facilitators.

**Table 3.** Perceived categories of COVID-19 exacerbating and inhibiting factors. Kaiser-Mayer-Olkin measure of sampling adequacy (KMO=53%) and degrees of freedom (*df*=10).

Perceived factors about COVID-19	Principal components and factor loading scores		Descriptive statistics (N=536)	
	Exacerbating factors	Inhibiting factors	n (%)	95% CI
Behavioral nonadherence^a^	0.71	—^b^	335 (62.5)	58.3-66.5
Lack of enabling environment^c^	0.71	—	494 (92.2)	89.6-94.2
Myths^d^	—	0.70	425 (79.3)	75.6-82.5
False assurances^e^	—	0.32	216 (40.3)	36.2-44.5
Engaged in precautions^f^	—	0.71	250 (46.6)	42.4-50.9

a Behavioral nonadherence: People fear stigma and bias related to COVID-19, people still use crowded transportation means, people with flu-like symptoms are not well screened for COVID-19, people refuse to take the COVID-19 vaccine, and people still hug and shake each other’s hands while greeting.

b Not applicable.

c Lack of enabling environment: People do not stay at home for economic and social reasons, people still live and work in a very crowded condition, people do not have personal protective equipment like face masks, and people do not have access to water or hand rub alcohol or sanitizers.

d Myths: The weather we live in is too hot for coronavirus to survive, we believe we have traditional medicine against COVID-19, and we are religious enough to control COVID-19.

e False assurances: We live far away from COVID-19’s rampant areas and COVID-19 is not risky or exaggerated.

f Engaged in precautions: Engaged in standard precaution measures for COVID-19 and vaccinated against COVID-19.

The table also includes categories and lists of perceived COVID-19 inhibitors as well as their respective prevalence. EFA produced 3 major categories of perceived factors that inhibited COVID-19. Two of the three categories were incorrectly identified (myths and false assurances), whereas one was correctly identified as an inhibitor. The myths category included factors that are thought to inhibit the virus but have not been scientifically proven. In this case, the myths included perceived religiosity (perceiving oneself as an effective religious man or woman in dealing with challenges), people’s perceived confidence in owning effective traditional medicines that were not clinically confirmed, and living in hot weather, with a factor loading score of 0.70.

The second category of perceived inhibitors, which were frequently associated with false assurances that people were immune to COVID-19. With factor loading scores of 0.32, the category consisted of 2 main beliefs: we live far away from COVID-19 hot spot areas and there have been no locally reported COVID-19 cases so far. The beliefs appeared to be false assurances in that people perceive themselves to be living outside a risk zone, giving the impression of invulnerability.

With a factor score loading of 0.71, the third, promotable category of perceived inhibitors concerned people who had taken standard precautions and were vaccinated against COVID-19. Factors related to the 3 categories explained 20.1% (108/532) of the variance in perceived inhibitors, indicating the presence of several other unreported myths and unhealthy beliefs that require further investigation.

### Risk Labeling

The COVID-19 risk labels and groups are presented in Table 4. People with underlying illnesses (118/536, 22%) and older adults (351/536, 65.5%) were perceived by the community to be at a high risk of COVID-19.

**Table 4.** Perceived COVID-19 risk groups and labels (N=536).

Perceived high-risk groups	Descriptive statistics, n (%)
Children (aged 0‐9 years)	38 (7.1)
Adolescents (aged 10-15 years)	3 (0.6)
Youths (aged 16‐29 years)	2 (0.4)
Adults (aged 30‐50 years)	11 (2.1)
Older persons (aged 51 years or more)	351 (65.5)
Pregnant women	9 (1.7)
Health workers	4 (0.7)
People with underlying chronic illness (eg, diabetes, hypertension, and cancer)	118 (22)

### Distribution of Perceived Exacerbating Factors

Table 5 illustrates the relationships between sociodemographic characteristics, behavioral nonadherence, and lack of an enabling environment in relation to COVID-19. The mean percentages of behavioral nonadherence and lack of an enabling environment as facilitators of virus spread were both high, at 62.5% (335/536) and 92.2% (494/536), respectively.

**Table 5.** Distribution of perceived exacerbating factors among the study participants.

Variables	Behavioral nonadherence^a^				Lack of enabling environment^b^			
	% Mean (SE)	*t* test (*df*=534)	*F* test (*df*=535)	*P* value	% Mean (SE)	*t* test (*df*=534)	*F* test (*df*=535)	*P* value
Overall	62.5 (2.1)				92.2 (1.2)			
**Sex**		–1.20		.12		2.78		.003^c^
Male	60.4 (2.8)				94.9 (1.2)			
Female	65.5 (3.2)				88.3 (2.2)			
**Age (years)**		–0.39		.35		0.51		.31
<40	62.1 (2.3)				92.4 (1.5)			
≥40	64.4 (5.2)				90.8 (3.1)			
**Education**			1.37	.23			3.55	.003^c^
Cannot read and write	50.0 (15.3)				80.0 (8.4)			
Read and write	63.2 (4.5)				86.8 (2.5)			
Primary (grade 1‐8)	55.1 (5.5)				87.2 (3.0)			
High school (grade 9‐12)	58.8 (3.9)				92.8 (2.1)			
Diploma or level I-IV	70.2 (4.7)				97.1 (2.6)			
First degree (BSc or BA) and above	67.5 (5.5)				98.7 (3.0)			
**Marital status**			0.24	.92			4.12	.003^c^
Single (unmarried)	63.2 (3.7)				94.8 (2.0)			
Married	62.1 (2.7)				90.7 (1.5)			
Divorced	53.8 (13.5)				92.3 (7.5)			
Widowed	75.0 (24.3)				75.0 (13.4)			
Engaged	70.0 (15.5)				—^d^			
**Employment**			3.33	.01^c^			0.75	.56
Private or business	64.4 (2.9)				90.8 (1.7)			
Daily laborer	48.0 (5.5)				90.7 (3.1)			
Government employee	60.0 (5.4)				96.3 (3.1)			
Student	82.4 (8.2)				94.1 (4.6)			
No job	64.0 (5.2)				93.0 (2.9)			
**Religion**			0.34	.71			0.35	.71
Muslim	62.9 (2.2)				92.0 (1.4)			
Orthodox	59.3 (9.3)				92.6 (6.2)			
Protestant	50.0 (17.2)				—			
**Place of origin**		1.89		.03^c^		–1.52		.06
Somali region	64.5 (2.3)				91.3 (1.4)			
Other regions	54.9 (4.7)				95.6 (1.9)			
**Internet connection mostly used to get information about COVID-19**			1.03	.36			7.68	<.001^c^
Cellular data (telle)	65.2 (2.9)				94.8 (1.6)			
Wireless Wi-Fi	56.5 (7.1)				—			
Do not use internet	60.1 (3.4)				86.7 (1.8)			
**Monthly income**		–2.84		.002^c^		0.2		.42
<5000 ETB^e^ (US $93)	55.2 (3.4)				92.4 (1.8)			
≥5000 ETB (US $93)	67.6 (2.6)				91.9 (1.5)			
**Source of information**			1.31	.25			6.84	<.001
Official websites	50.0 (34.1)				—			
Television	60.1 (3.2)				92.1 (1.7)			
Radio	50.0 (12.1)				93.8 (6.5)			
Friends or neighbors	55.2 (6.3)				75.8 (3.4)			
Social media (Facebook, Twitter, YouTube, etc)	68.9 (3.5)				97.9 (1.9)			
Health workers	61.1 (8.1)				86.1 (4.4)			

a Behavioral nonadherence: People fear stigma and bias related to COVID-19, people still use crowded transportation means, people with flu-like symptoms are not well screened for COVID-19, people refuse to take the COVID-19 vaccine, and people still hug and shake each other’s hands while greeting.

b Lack of enabling environment: People do not stay at home for economic and social reasons, people still live and work in a very crowded condition, people do not have personal protective equipment like face masks, and people do not have access to water or hand rub alcohol or sanitizers.

c Statistically significant at *P*<.05 (two-tailed).

d Not applicable.

e ETB: Ethiopian Birr.

In terms of behavioral nonadherence, respondents’ employment, region, and income showed significant mean percentage differences. Students had the highest mean percentage of behavioral nonadherence (442/536, 82.4%; *P*=.01) when it came to employment. Respondents of Somali origin and those with an income of more than 5000 ETB (US $93) had the highest mean percentages of behavioral nonadherence (346/536, 64.6%; *P*=.03) and (362/536, 67.5%; *P*=.003), respectively. There was no difference in behavioral nonadherence by age, gender, education, marital status, religion, primary internet source, or primary information source for COVID-19.

Similarly, the lack of an enabling environment that was perceived as a facilitator of the spread of the virus was more prevalent among male respondents (509/536, 95%; *P*=.003), respondents whose education status was first degree and above (529/536, 98.7%; *P*=.003), respondents who were single and not married (508/536, 94.8%; *P*=.001), those who used cellular data as their primary source of the internet (508/536, 94.8%; *P*=.001), and those who used social media as their primary source of information (525/536, 97.9%; *P*<.001). There was no difference in behavioral nonadherence by age, employment, religion, place of origin, and monthly income.

### Distribution of Perceived Inhibiting Factors

The factors deemed to hinder the transmission and control of the virus exhibited considerable sociodemographic disparities (Table 6). The highest perceived inhibitor to control the spread of COVID-19 was seen to be myth (425/536, 79.3%), followed by preventive measures (250/536, 46.6%) and false guarantees (214/536, 39.9%).

**Table 6.** Distribution of perceived inhibiting factors among the study participants.

Variables	Myth^a^				False assurance^b^				Engaged in precaution^c^			
	% Mean (SE)	*t* test (*df*=534)	*F* test (*df*=535)	*P* value	% Mean (SE)	*t* test (*df*=534)	*F* test (*df*=535)	*P* value	% Mean (SE)	*t* test (*df*=534)	*F* test (*df*=535)	*P* value
Overall	79.3 (1.8)				40.3 (2.1)				46.6 (2.2)			
**Sex**		2.79		.003^d^		0.33		.37		–0.17		.43
Male	83.4 (2.1)				40.9 (2.8)				46.3 (2.8)			
Female	73.4 (3.0)				39.4 (3.3)				47.1 (3.4)			
**Age (years)**		–0.58		.28		–0.70		.76		0.73		.23
<40	78.8 (1.9)				39.6 (2.3)				47.0 (2.3)			
≥40	81.6 (4.2)				43.7 (5.3)				44.8 (5.4)			
**Education**			7.37	<.001^d^			2.12	.06			3.72	.003^d^
Cannot read and write	90.0 (12.5)				50.0 (15.4)				60.0 (15.6)			
Read and write	79.8 (3.7)				46.5 (4.6)				40.4 (4.6)			
Primary (grade 1‐8)	60.3 (4.5)				30.8 (5.5)				47.4 (5.6)			
High school (grade 9‐12)	74.5 (3.2)				46.4 (3.9)				36.6 (4.0)			
Diploma or level I-IV	88.5 (3.9)				37.5 (4.8)				58.7 (4.8)			
First degree (BSc or BA) and above	93.5 (4.5)				31.2 (5.6)				57.1 (5.6)			
**Marital status**			1.42	.23			0.8	.52			0.94	.44
Single (unmarried)	83.3 (3.1)				36.8 (3.7)				47.7 (3.8)			
Married	77.6 (2.2)				41.8 (2.7)				45.7 (2.7)			
Divorced	76.9 (11.2)				38.4 (13.6)				61.5 (13.9)			
Widowed	—^e^				75.0 (24.6)				75.0 (25.0)			
Engaged (in relationship)	60.0 (12.8)				40.0 (15.5)				30.0 (15.8)			
**Employment**			2.51	.041^d^			4.81	<.001^d^			0.47	.76
Private or business	82.0 (2.5)				49.0 (3.0)				45.2 (3.1)			
Daily laborer	66.7 (4.7)				30.7 (5.6)				48.0 (5.8)			
Government employee	83.8 (4.5)				38.8 (5.4)				43.8 (5.6)			
Student	82.4 (6.9)				32.4 (8.3)				55.9 (8.6)			
No job	76.7 (4.3)				26.7 (5.2)				48.8 (5.4)			
**Religion**			0.21	.81			0.95	.39			0.17	.85
Muslim	79.0 (1.8)				39.7 (2.2)				46.9 (2.2)			
Orthodox	81.5 (7.8)				44.4 (9.4)				44.4 (9.6)			
Protestant	87.5 (14.4)				62.5 (17.4)				37.5 (17.7)			
**Place of origin**			0.68	.25		–1.82		.03^d^		0.99		.16
Somali region	79.9 (2.0)				38.3 (2.4)				47.8 (2.4)			
Other regions^f^	77.5 (4.0)				47.8 (4.7)				42.5 (4.7)			
**Internet connection mostly used to get information about COVID-19**			0.46	.63			5.37	.005^d^			4.1	.02^d^
Cellular data (telle)	78.7 (2.9)				36.9 (2.9)				49.8 (2.9)			
Wireless Wi-Fi	84.8 (7.2)				26.1 (7.2)				58.7 (7.3)			
Do not use internet	78.8 (3.4)				48.3 (3.4)				39.4 (3.5)			
**Monthly income**		–0.02		.49		3.74		<.001^d^		–4.29		<.001^d^
<5000 ETB^g^ (US $93)	79.2 (2.8)				50.0 (3.4)				35.4 (3.3)			
≥5000 ETB (US $93)	79.3 (2.3)				33.9 (2.6)				54.0 (2.8)			
**Source of information**			2.32	.042^d^			3.18	.008^d^			2.75	.02^d^
Official website	—				50.0 (34.4)				50.0 (35.0)			
Television	77.2 (2.7)				36.8 (3.2)				45.6 (3.3)			
Radio	56.3 (10.1)				31.3 (12.1)				18.8 (12.4)			
Friends and neighbors	74.1 (5.3)				62.1 (6.4)				32.8 (6.5)			
Social media^h^	85.2 (2.9)				36.7 (3.5)				53.6 (3.5)			
Health workers	77.8 (6.7)				50.0 (8.1)				50.0 (8.3)			

a Myths: The weather we live in is too hot for coronavirus to survive, we believe we have traditional medicine against COVID-19, and we are religious enough to control COVID-19.

b False assurances: We live far away from COVID-19’s rampant areas, and COVID-19 is not risky or exaggerated.

c Engaged in precautions: Engaged in standard precautions for COVID-19 and vaccinated against COVID-19.

d Statistically significant at *P*<.05 (two-tailed).

e Not applicable.

f Other regions include Addis Ababa, Afar, Amhara, Oromia, southern nations and nationalities, and Tigray.

g ETB: Ethiopian Birr.

h Facebook, Twitter, YouTube, etc.

Male respondents (447/536, 83.4%; *P*=.003), those with a first degree or higher (501/536, 93.5%; *P*<.001), government employees (449/536, 83.8%; *P*=.041), and those who used social media as their primary source of information (457/536, 85.3%; *P*=.042) had a significantly higher prevalence of myths. There was no difference in myth prevalence by age, marital status, religion, place of origin, primary internet source, or monthly income.

False assurances were also more common among those who owned or ran a private business (263/536, 49.1%; *P*=.001), non-Somalis (256/536, 47.8%; *P*=.03), noninternet users (259/536, 48.3%; *P*=.005), those with monthly income less than 5000 ETB (US $93; 268/536, 50%; *P*=.001), and those who get COVID-19 information from friends and neighbors (333/536, 62.1%; *P*<.001). There was no significant variation in the prevalence of false assurance by age, sex, education, marital status, religion, employment, or primary information source for COVID-19.

Likewise, participants who were unable to read and write, those who used Wi-Fi as their primary source of internet, those who earned more than 5000 ETB (US $93), and those who used social media as their primary source of COVID-19 information were more likely to take precautionary measures than their counterparts. There was no significant variation in the prevalence of precautionary measures according to age, sex, marital status, religion, and employment.

## Discussion

### Principal Findings

This study produced relevant findings regarding community perceptions of the factors that facilitate and inhibit the spread of COVID-19, risk labeling, and information needs in a border town in the Somali region. The perceived factors were classified into the following major categories: behavioral adherence, a lack of enabling environmental conditions, and risk labeling.

Factors perceived to exacerbate virus spread were grouped into 2 thematic categories: behavioral nonadherence (335/536, 62.5%) and a lack of enabling environmental conditions (494/536, 92.2%). The study revealed a concerning lack of awareness and adherence with 80% (429/536) of respondents noting that individuals continue to disregard social distancing. Additionally, 38.1% (204/536) of those surveyed indicated that people fear the stigma associated with the virus, while 32.5% (174/536) expressed unwillingness to receive the COVID-19 vaccine. These results highlight a significant gap in the understanding of and compliance with preventive measures.

The use of crowded transportation modes was not only due to a lack of alternatives but also associated with behavioral nonadherence. Fears, threat appraisals, and worries have consistently been linked to increased adherence in COVID-19 protective behaviors [11,12]. An Iranian study found that increased fear predicted less adherence [13]. This study shows that clear communication of risk could increase participation in protective behaviors, especially among disengaged groups.

Conversely, people could not stay at home for economic and social reasons (494/536, 92.2%); they did not have access to face masks, water, or sanitizers (29/536, 5.4%); and they still lived and worked in crowded conditions (403/536, 75.2%). According to behavioral and communication theories, people’s perceived lack of resources has a negative impact on their actual practices [14]. Despite this, the high prevalence of perceived facilitators indicates 2 major and urgent issues. First, it indicates a strong effort to reduce behavioral nonadherence and a lack of enablers that facilitate viral spread. Second, even a higher perceived lack of enabling conditions appears concerning because it may lead people to externalize their ability to control the virus while ignoring their own efforts.

Poverty and livelihood issues are major challenges in COVID-19 prevention in low-income countries. This is because COVID-19 has impacted people’s livelihoods and income opportunities, resulting in food insecurity [15]. Most people in Tog Wajaale are low-income earners who work in the informal sector and rely on daily wages to feed their families and cover their related expenses. As most of them struggle to earn a living, COVID-19 has added to their difficulties. As a result, they are confronted with the harsh reality of contracting the virus while attempting to survive. The observation results show that despite the lockdown, daily laborers in the construction sector, informal businesses, and street vendors continued their daily activities [16].

Factors perceived as inhibitors of viral spread were divided into 3 categories: false assurances (216/536, 40.3%), myths (425/536, 79.3%), and standard precautions (250/536, 46.6%). False assurances were perceptions of invulnerability, characterized by people believing that they were living outside the COVID-19 risk zone. The 2 main false assurances in this study were the perception of COVID-19 as less risky or exaggerated and residence far away from the COVID-19 rampant areas. Myths in this study included religious effectiveness (425/536, 79.3%), living in hot weather (358/536, 66.8%), and traditional medicine (36/536, 6.7%). The WHO myth busters list the majority of the misconceptions presented in this study, indicating that they were universally held during the pandemic [17]. Evidence suggests that myths or misperceptions, such as denial of presence and misperceived causes, transmission modes, and prevention methods, can prevent and control efforts during pandemics of HIV, Zika virus, yellow fever, and Ebola unless traced and addressed [18-21].

Culture and religion are important components of Somalis’ social and moral fabrics. As a result, cultural and religious misunderstandings as well as misinformation about the virus are among the factors contributing to complacency when it comes to prevention measures. According to a study conducted in northern Ethiopia, people believe that COVID-19 is a “punishment from God” or “Allah” for people’s immoral acts, and that the power of prayer can prevent the pandemic’s severe effects [16].

The magnitude of correctly perceived factor engagement in standard precautionary measures (134/536, 25%) and vaccination against COVID-19 (160/536, 29.9%) was too low, necessitating hard work to promote this perception, until a larger segment of the community embraced an accurate reason for virus protection.

Respondents’ employment, region, and income all showed significant mean percentage differences in behavioral nonadherence. When it came to employment, students had the highest mean percentage of behavioral nonadherence (442/536, 82.5%). Respondents of Somali origin and those with an income of more than 5000 ETB (US $93) had the highest mean percentages of behavioral nonadherence (64.6%, 346/536 and 67.5%, 362/536, respectively). Students were most likely to be of younger age, and being younger was found to be a predicator of nonadherence to preventive behavior [22]. Regular communication regarding SARS-CoV-2 emphasizes that COVID-19 mostly threatens the older population. This could send a false message of safety to the younger generations. Younger individuals’ lower adherence may be related to their perceived lower vulnerability and propensity to make impulsive decisions, take greater risks to gain emotional and social stimulation (such as attending social gatherings), and fail to recognize the long-term effects of their actions [23].

Similarly, almost 60.1% (322/536) of the communities surveyed are reliant on cross-border commerce and daily labor, and 20% (107/536) of them live on less than US $100 per month. Thus, they are driven to leave their homes, even during the COVID-19 epidemic, to sustain their families. Within the Somali population, males typically serve as primary income earners and feel obligated to venture outside their homes, even during the COVID-19 pandemic, to earn money to sustain their families. This observation aligns with a study conducted in Somalia, which also noted that Somali men tend to disregard COVID-19 preventive protocols [24].

Obtaining COVID-19 information from official sources (radio, television, and government announcements) increased the likelihood of having a high adherence score, whereas obtaining it from social media decreased the likelihood of having a high adherence score. This highlights the role of misinformation in determining adherence to scientifically proven COVID-19 prevention measures [25]. In addition, a previous study showed that social media users tend to be more extroverted, suggesting that these people may find it more challenging to stay at home and observe COVID-19 preventive measures than introverted non–social media users [26].

### Limitations

First, the cross-sectional design does not provide conclusive conclusions about causality, and social desirability bias may skew the self-reported data. Second, only one border crossing town in parts of the Somali region was surveyed, and the results of this study may not reﬂect the perceptions of other towns in the Somali region and Ethiopia. A similar study could be extended to other border-crossing towns in the Somali region and Ethiopia.

### Recommendations

There is a need for local activities that encourage participation in standard precautions by addressing locally perceived hurdles and fostering a sense of shared responsibility and community ownership during the fight against COVID-19. Consequently, RCCE efforts in Tog Wajaale should (1) consider sociodemographic variations in myths and false assurances, (2) investigate more beliefs that could facilitate or inhibit the spread of the virus, and (3) design local initiatives that enhance community ownership of tasks associated with controlling the virus, thus supporting and advocating engagement in standard precautionary measures. That is, using community and religious leaders to spread correct information, model precautionary behaviors, and organize COVID-19–related activities.

### Conclusions

The results suggest that extensive efforts in RCCE are necessary. This conclusion is supported by several factors. First, only 44.6% (239/536) of individuals correctly engaged in precautionary measures. Second, there was a high perceived prevalence of behavioral nonadherence and a shortage of essential resources in the COVID-19 response. Lastly, numerous misconceptions and false reassurances were identified among the population. Thus, understanding the behavior of cross-border communities is crucial for developing effective, contextually appropriate RCCE strategies to mitigate COVID-19 risk in border communities.

## Abbreviations

EFA: exploratory factor analysis
ETB: Ethiopian Birr
KMO: Kaiser-Mayer-Olkin measure
PPS: probability proportional to size
RCCE: risk communication and community engagement
WHO: World Health Organization
